# Translation, cultural adaptation, and validation of the Family Dermatology Life Quality Index instrument into the Brazilian Portuguese language (FDLQI-BRA)^⋆^

**DOI:** 10.1016/j.abd.2024.02.005

**Published:** 2024-08-07

**Authors:** Maria Laura Malzoni Souza, Hélio Amante Miot, José Eduardo Martinez

**Affiliations:** a Department of Dermatology, Pontifícia Universidade Católica de São Paulo, Sorocaba, SP, Brazil; b Department of Infectology, Dermatology, Imaging Diagnosis and Radiotherapy, Faculty of Medicine, Universidade Estadual Paulista, Botucatu, SP, Brazil

Dear Editor,

Dermatological diseases are very prevalent; however, despite generally showing low mortality, they can have high morbidity and impact on Quality of Life (QoL). Moreover, living with people with dermatoses can reflect anguish, requiring care and financial expenses. Therefore, dermatological diseases can interfere with family dynamics. The impact of the disease on patients QoL is called “primary impact”, and when it involves family members and/or cohabitants, it is called “secondary impact”.^1^ Understanding the difficulties faced by family members is essential for creating educational programs and planning support to promote the QoL of those who live with dermatological patients.

The Family Dermatology Life Quality Index (FDLQI) is a generic, self-completed instrument for assessing the secondary impact on people living with patients with dermatological diseases. It consists of ten items with responses graded on a Likert-type scale, ranging from 0 (“not at all”) to 3 (“very much”), considering physical aspects (fatigue and physical overload), psychological and social aspects, as well as those related to personal, work and financial relationships.^2^ It showed high internal consistency for several languages ​​such as English (original), Japanese, and Persian.^3^, ^4^ It was developed at Cardiff University (Wales), by the same group that developed the Dermatology Life Quality Index (DLQI). There are no other generic instruments for assessing the QoL of people living with skin diseases. However, there are specific questionnaires available, such as the Psoriasis Family Index (PFI) and the Family Dermatitis Impact (FDI), both translated and validated in Brazil.^5^, ^6^

A methodological study was carried out aiming at translating, culturally adapting, and validating the FDLQI into Brazilian Portuguese (FDLQI-BRA). The project was approved by the institutional ethics committee and consent was obtained from the participants.

After obtaining the authors’ authorization, the translation into Portuguese and back-translation were carried out. For this stage, four experts fluent in Portuguese and English, and one non-expert, generated a consensual translated version, the back-translation of which was approved by the instrument authors.^7^

For the cultural adaptation, ten family members of patients with dermatological diseases answered a questionnaire about the clarity of the language used, the adequacy of the linguistic terms adopted, the applicability of the instrument, and its relevance in dermatological clinical practice. This stage generated the final Brazilian version: FDLQI-BRA (Supplementary Materials 1 and 2).

For content validation, five experienced dermatologists were selected and asked to evaluate the items according to their relevance and pertinence. This assessment was carried out using a Likert scale, graded from 1 to 5 – with 1 being irrelevant/not very pertinent; and 5 very relevant/pertinent.^7^ The calculation of the Content Validation Index (CVI) resulted in scores greater than or equal to 0.8 in most items, with the exception of item 6 (“recreation and leisure”), with 0.6.^8^

For the FDLQI-BRA validation phase, 111 participants, aged 18 years or older, who lived in the patient's home were included. The non-inclusion criteria were family members with complicated or uncontrolled comorbidities, which could interfere with their QoL. The initial sample size was based on the need for around ten participants for each item of the unidimensional instrument being validated, totaling at least 100 family members.^9^

The participants were recruited both in the outpatient clinics of the Dermatology Service at Botucatu Faculty of Medicine, Unesp, Botucatu, São Paulo, Brazil, and in the researcher's private office (Sorocaba-SP). There was no selection by disease.

A subgroup of 20 family members answered the questionnaire again every three to ten days, although the patient's dermatological condition was maintained, to estimate the temporal stability of the instrument. Another subgroup of 20 family members answered the questionnaire every 30 to 60 days, after observation of clinical improvement in the dermatological condition, to estimate instrument responsiveness.

Internal consistency was assessed using the Cronbach-α coefficient and the correlation between the instrument items was calculated using Spearman’s coefficient. Temporal stability was estimated using the intraclass correlation coefficient, and responsiveness using Wilcoxon’s test.

The family members’ demographic findings are shown in Table 1 and the patients’ dermatological diagnoses are described in Table 2. All questionnaires were completely filled out, with no doubts about item composition.

**Table 1:** Main data from family members who participated in the FDLQI-BRA validation stage (n = 111).

Variables		Results
Family relationship	Parents	54 (49%)
	Partners	30 (27%)
	Siblings	12 (11%)
	Children	8 (7.5%)
	Grandparents	7 (6.5%)
Gender	Female	79 (71%)
	Male	32 (29%)
Age, years	Mean (SD)	42 (13)
Level of schooling	Elementary School	31 (27%)
	High School	40 (36%)
	Higher Education	40 (36%)
FDLQI-BRA	Median (p25-p75)	6 (4‒12)

SD, Standard deviation; p25-p75, First and third quartiles.

**Table 2:** Dermatological diagnoses of the patients (n = 111).

Diagnosis	Number of patients	%
Acne	18	16%
Atopic dermatitis	11	10%
Psoriasis	7	6%
Skin cancer	7	6%
Viral wart	6	5%
Contact dermatitis	4	4%
Actinic keratosis	3	3%
Melasma	3	3%
Rosacea	3	3%
Alopecia areata	3	3%
Seborrheic dermatitis	3	3%
Lower limb ulcer	3	3%
Androgenetic alopecia	2	2%
Lupus	2	2%
Dyshidrosis	2	2%
Molluscum contagiosum	2	2%
Others	32	27%

The FDLQI-BRA scores ranged from 0 to 27. The distribution of scores was asymmetric for most items (Fig. 1). Concomitantly, items F5 (“social life”), F6 (“recreation/leisure”), and F9 (“work/study”) showed more than 60% of the occurrences classified as “not at all”, generating a “floor effect”.^10^

**Fig. 1:**
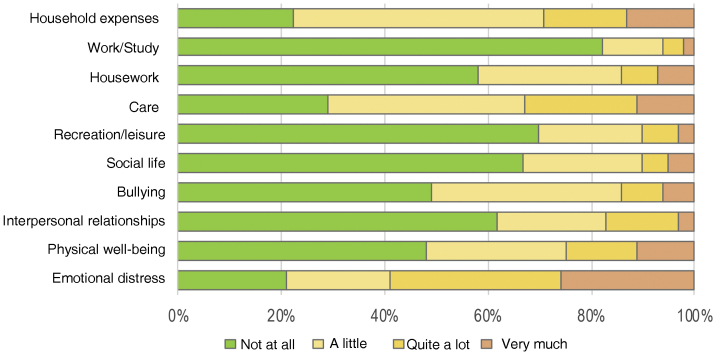
Distribution of the Brazilian Portuguese version of the Family Dermatology Life Quality Index scores in each item (n = 111).

The analysis of the overall internal consistency of the FDLQI-BRA (Cronbach-α coefficient) resulted in 0.855 (95% CI: 0.818‒0.887), proving to be adequate for this instrument.

Horn's parallel analysis indicated unidimensionality for the FDLQI-BRA. The KMO coefficient was 0.803 (p < 0.01), indicating sample adequacy for the exploratory factor analysis. The variance of the latent variable explained by the unidimensional factor was 53.6%, and the item-item and item-total correlation matrix resulted in the coefficients shown in Table 3.

**Table 3:** Item-item and item-total correlation (Spearman's rho) of the FDLQI-BRA (n = 111).

	F1	F2	F3	F4	F5	F6	F7	F8	F9	F10
F2	0.563									
F3	0.283	0.569								
F4	0.323	0.315	0.395							
F5	0.258	0.363	0.508	0.316						
F6	0.308	0.470	0.400	0.177	0.50					
F7	0.345	0.479	0.190	0.277	0.220	0.150				
F8	0.206	0.352	0.318	0.341	0.381	0.249	0.440			
F9	0.354	0.379	0.377	0.154	0.377	0.336	0.153	0.171		
F10	0.244	0.561	0.366	0.211	0.220	0.305	0.404	0.404	0.177	
FDLQI-BRA	0.689	0.825	0.641	0.564	0.566	0.564	0.601	0.576	0.451	0.628

FDLQI-BRA, Brazilian Portuguese version of the Family Dermatology Life Quality Index.

Regarding temporal stability, the intraclass correlation coefficient for complete agreement was 0.998 (p < 0.05). As for the analysis of responsiveness, or sensitivity of the instrument to change, there was a reduction in the FDLQI-BRA scores after treatments were implemented and clinical improvement of the disease (p < 0.01), with the test values ​​showing a mean (SD) of 11 (4.6), and the retest values, 4 (3.7).

The psychometric analysis of the FDLQI-BRA produced results that guaranteed content, structural, and construction validity, temporal stability and responsiveness, indicating it is a valid instrument for clinical use in Brazil.

The distribution of scores and internal consistency were similar to other international validations, which maximized their interpretability.^2^, ^3^ However, the instrument presented some reservations regarding the behavior of some items that were poorly correlated with each other, especially those influenced by the “floor” effect of some covered topics, since work, sports/leisure and social life may not be homogeneous in the population.^10^

It should be noted that the family sample comprised, for the most part, women and mothers, with data from men being restricted. Moreover, as the questionnaires were applied after outpatient consultations, and not in emergency departments and inpatient units, a majority of patients with mild to moderate impact on QoL were included. Finally, there is no other reference instrument for concurrent criterion validation.^7^, ^11^

Given the extent of the impact of dermatological diseases on QoL, some authors have already demonstrated an increase in the frequency of psychiatric diseases in dermatological patients, as well as an increased risk of suicide, with dermatological diseases often causing greater concern in individuals than certain systemic diseases.^12^

In view of this, the understanding, improvement and development of QoL assessment instruments are fundamental for dermatological clinical practice, for therapeutic trials and for the improvement of educational policies for the population.

In conclusion, a Brazilian Portuguese version of the FDLQI was translated and adapted, which proved to be valid and consistent (FDLQI-BRA).

## Financial support

None declared.

## Authors' contributions

Maria Laura Malzoni Souza: Design and planning of the study; critical review of the literature; collection of data; drafting and editing of the manuscript.

Hélio Amante Miot: Design and planning of the study, collection and analysis of data, drafting and editing of the manuscript and approval of the final version of the manuscript.

José Eduardo Martinez: Design and planning of the study; effective participation in research orientation; approval of the final version of the manuscript.

## Conflicts of interest

None declared.
