# Supplementary material for: Translation, cultural adaptation, and validation of the Family Dermatology Life Quality Index instrument into the Brazilian Portuguese language (FDLQI-BRA)
Source: An Bras Dermatol. 2024 Aug 7;99(6):939–42. doi: 10.1016/j.abd.2024.02.005 (PMC11551233; doi:10.1016/j.abd.2024.02.005)
Supplement: Supplementary file 1 [file mmc1.docx]

ABD-D-23-00658_Supplementary Material

**Supplementary Material 1** Original version of the FDLQI (in English): https://www.cardiff.ac.uk/__data/assets/word_doc/0006/1340628/FDLQI.docx.

**Supplementary Material 2** Brazilian Portuguese Version of the FDLQI-BRA: https://doi.org/10.17632/kgtwnz4p2t.1.
